# Influence of Chemical-Modified Cotton on Thermal Properties of Flexible Polyurethane Foams and Associated Fire Hazard

**DOI:** 10.3390/polym18121432

**Published:** 2026-06-08

**Authors:** Arkadiusz Głowacki, Przemysław Rybiński, Witold Żukowski, Anna Zawierucha, Monika Żelezik, Salaeh Subhan

**Affiliations:** 1 Institute of Chemistry, Jan Kochanowski University, 25-406 Kielce, Poland; annazawierucha.011@gmail.com; 2 Faculty of Chemical Engineering and Technology, Cracow University of Technology, Warszawska 24, 31-155 Kraków, Poland; witold.zukowski@pk.edu.pl; 3 Institute of Geography and Environmental Sciences, Jan Kochanowski University, 25-406 Kielce, Poland; monika.zelezik@ujk.edu.pl; 4 Department of Rubber Technology and Polymer Science, Faculty of Science and Technology, Prince of Songkla University, Pattani 94000, Thailand; subhan.s@psu.ac.th

**Keywords:** polyurethane foams, surface morphology, thermal properties, flammability, smoke emissions, toxicity

## Abstract

In this study, a new approach to improving the fire resistance of flexible polyurethane (PUR) foams is presented, based on the incorporation of cotton chemically modified with boron compounds into the polyurethane matrix. The developed system was additionally modified with melamine polyphosphate (MPP). The effects of the applied modifications on the morphology and chemical structure of the PUR composites were investigated using scanning electron microscopy and infrared spectroscopy. Thermal stability was evaluated by thermogravimetric analysis, whereas fire hazard was assessed using cone calorimetry and a smoke optical density chamber. The toxicometric index (WLC_50SM_) was determined using a coupled TG-Omega 5 gas analyzer system. The results provide insight into the mechanism responsible for reducing flammability and limiting the emission of toxic combustion and thermal decomposition products through the modification of PUR foams with chemically modified cotton in combination with MPP. It was observed that, during the combustion of the developed PUR composites, the addition of cotton promotes the formation of a three-dimensional spatial network, which substantially limits heat release and the emission of toxic combustion products. Consequently, the composites exhibited a reduction in heat release of up to 67% in terms of HRR_MAX_, together with decreased production of HCN and CO. Nevertheless, the formation of a protective carbon layer contributed to an increase in smoke optical density, which was associated with increased CO_2_ emission. Overall, this work demonstrates the development of a new synergistic system capable of reducing both the flammability and toxicity of flexible PUR foams.

## 1. Introduction

Polyurethane (PUR) materials are widely used in everyday life as well as in numerous industrial sectors, including the construction, automotive and furniture industries, where they serve as insulation, structural, and upholstery materials [1,2,3,4,5]. The broad range of PUR applications is primarily associated with their excellent physicochemical properties, such as low thermal conductivity, low density, good abrasion resistance, and favourable cushioning performance. Another important advantage of polyurethane materials is the possibility of tailoring their properties through the selection of raw materials, adjustment of the reagent ratio, incorporation of modifying additives or fillers, and optimization of processing conditions. Depending on their chemical composition and manufacturing route, PUR materials may exhibit high mechanical strength, good damping properties, enhanced resistance to physical and chemical factors, and resistance to organic solvents and oils [6,7,8].

Despite these numerous advantages, polyurethane materials also exhibit significant limitations in practical applications. One of the most important drawbacks is their high susceptibility to ignition and the intense course of thermal decomposition, which considerably limits their safe use, particularly in construction and transportation applications [9]. The combustion of PUR foams is also accompanied by significant heat release, smoke generation, and the emission of toxic thermal decomposition and combustion products, including carbon monoxide (CO), carbon dioxide (CO_2_), and, at elevated temperatures, nitrogen oxides (NO_X_) and hydrogen cyanide (HCN). These gases may cause severe disturbances in the respiratory, cardiovascular and nervous systems. Under fire conditions, they therefore constitute one of the major threats to human health and life [10,11,12,13,14].

In recent years, particular attention has been focused on the modification of polyurethanes using additive or reactive flame retardants, including synergistic systems combining phosphorus-, nitrogen-, and boron-containing compounds with additives of organic or mineral origin. This approach enables not only the suppression of the thermal decomposition intensity of PUR materials but, more importantly, a reduction in their flammability through the formation of an insulating char layer on the material surface, which restricts mass and energy transfer between the sample and the flame [15,16,17,18].

Currently, increasing attention is being paid to the use of fibrous lignocellulosic raw materials as components of polymer composites, which is driven by both environmental and performance-related considerations. Natural fibres are regarded as an attractive alternative to conventional fillers and synthetic fibres due to their renewable nature, relatively low cost, low density, and favourable strength-to-weight ratio. For this reason, natural-fibre-reinforced composites are finding increasingly broad applications, particularly in the automotive, construction, and packaging sectors [19,20,21,22,23,24].

The effectiveness of natural fibres in polymer composites depends primarily on the type and chemical composition of the fibre, the method of surface treatment, and its compatibility with the polymer matrix.

Among natural fibres, cotton is of particular importance. Owing to its high cellulose content and low apparent density, it is increasingly regarded as an attractive component of polymer composites. Cellulose, which is rich in hydroxyl groups, favourably affects the surface reactivity of cotton fibres and enables their further chemical modification, which is of key importance in the design of functional materials [25,26,27,28,29].

An undeniable drawback of cotton-based materials is their high flammability. Cotton can burn completely in atmospheres with an oxygen concentration lower than that of ambient air. Therefore, current research efforts are aimed at reducing its flammability while simultaneously improving its thermal stability. Particularly interesting in this context is the modification of cotton with boron compounds, which are currently considered precursors of ceramic-like structures.

The aim of this study was to assess the effect of unmodified cotton and cotton previously modified with boron compounds, also in a synergistic system with melamine polyphosphate, on the properties of flexible polyurethane foams. The influence of the cotton–MPP system on the properties of PUR composites was analyzed. Particular attention was paid to the effects of cotton, including chemically modified cotton, also in combination with MPP, on the thermal stability, flammability, smoke-forming behaviour, and toxicity of gaseous degradation products generated from PUR composites.

## 2. Materials and Methods

### 2.1. Materials

The investigated material was a flexible polyurethane foam. The reference PUR material was prepared using an isocyanate, diphenylmethane diisocyanate (MDI) (BASF (Ludwigshafen am Rhein, Germany), IZO 135/158), and polyol, diethanolamine (BASF, Elastoflex W5165/140). Blowing agents and catalysts were incorporated into the polyol by the manufacturer. Unmodified cotton, cotton modified with boron compounds, and melamine polyphosphate (MPP) were used as PUR fillers.

#### 2.1.1. Preparation of Chemically Modified Cotton

The chemical modification of cotton was carried out using the sol–gel method with a SiO_2_/B system, in which disodium octaborate tetrahydrate was used as the boron-containing component. In the first stage, a solution containing the components of the SiO_2_/B system at a ratio of 1:6 was prepared and then subjected to acid-catalyzed hydrolysis using 0.1 M HCl to obtain a stable sol. Before modification, the cotton was dried at 40 °C for 24 h to remove moisture physically adsorbed on the fibre surface and to stabilize the material prior to the impregnation process. The cotton was then immersed in the prepared sol, and impregnation was carried out with stirring for 5 min to ensure the uniform wetting of the fibres and deposition of the modifying components on their surface. After impregnation, the material was drained and dried at 40 °C for 6 h. The entire process of impregnation, draining, and drying was repeated three times under the same conditions to increase the degree of deposition and improve the uniformity of the modifying layer on the cotton fiber surface.

#### 2.1.2. Preparation Procedure of PUR Composites

Flexible PUR foams were prepared by the polycondensation of the polyol with the isocyanate at an OH:NCO ratio of 2:1. The reference unmodified flexible PUR foam was obtained in a single-step reaction at room temperature (22–24 °C). The polyol and isocyanate compounds were introduced into a reaction vessel and mixed using a mechanical stirrer until the system foamed. The foamed system was poured into an appropriate test mould with a volume of 0.9 L. After synthesis, the obtained PUR foams were left in the open mould for approximately 60 min to stabilize its structure. The preparation of PUR composites involved the prior dispersion of the fillers in the polyol component. The fillers were added in the proportions listed in Table 1, and the mixtures were homogenized to obtain the most uniform filler distribution possible. Subsequently, the composite foams were synthesized according to the same procedure used for the unmodified PUR foam, with the only difference being the presence of the filler-containing polyol phase.

Before testing, the prepared composites were conditioned to constant mass at a temperature of 23 ± 2 °C and a relative humidity not exceeding 50 ± 5%, in accordance with the guidelines of ISO 291 [30]. The conditioning process was considered complete when the sample mass after 24 h showed no change greater than 0.1 g or 0.1% of the initial mass.

### 2.2. Methods

#### 2.2.1. Fourier-Transform Infrared Spectroscopy

Fourier-transform infrared spectroscopy was performed using a PerkinElmer Spectrum Two spectrometer (PerkinElmer, Waltham, MA, USA) equipped with an attenuated total reflectance (ATR) accessory with a diamond crystal mounted on a ZnSe plate. The spectra were recorded using Spectrum software 10.03.06, PerkinElmer (Waltham, MA, USA). Measurements were carried out in the mid-infrared region (MIR), within the range of 400–4000 cm^−1^, at a spectral resolution of 4 cm^−1^, with 4 scans collected for each measurement.

#### 2.2.2. Scanning Electron Microscopy

The morphology of the samples was examined by scanning electron microscopy using an Apreo 2 S LoVac microscope (Thermo Fisher Scientific, Waltham, MA, USA) equipped with UltraDry (Thermo Fisher Scientific, Waltham, MA, USA) and Octane Elect EDS detectors (EDAX, Ametek GmbH/Hitachi, Tokyo, Japan). The SEM observations were conducted at an accelerating voltage of 2 kV.

#### 2.2.3. Thermal Analysis

A thermogravimetric analysis of the fillers and PUR composites was performed using an STA 449 F3 Jupiter thermal analyzer (Netzsch, Selb, Germany). The measurements were conducted from 25 to 650 °C for the PUR composites, using samples with a mass of 5 ± 1 mg. The samples were placed in open Al_2_O_3_ crucibles. Measurements were performed in an oxygen/nitrogen atmosphere with gas flow rates of 20/40 µL/min, with a constant heating rate of 10 °C/min. The thermogravimetric curves were processed using Proteus Thermal Analysis 8.0.3 software, Netzsch (Selb, Germany). Based on the TG and DTG curves, the following thermal parameters were determined: temperature corresponding to 5% mass loss (T_5_), temperature corresponding to 50% mass loss (T_50_), the maximum thermal decomposition rate (dm/dt), the temperature of the maximum thermal decomposition rate (T_RMAX_), residue after thermal decomposition (P_TD_), and residue at 600 °C (P_600_).

#### 2.2.4. Cone Calorimetry

The flammability of the investigated materials under forced-combustion conditions was evaluated using a cone calorimeter supplied by Fire Testing Technology Ltd. (East Grinstead, UK). The measurements were performed in accordance with PN-EN ISO 5660 [31]. Samples with dimensions of 100 mm × 100 mm × 5 mm were placed horizontally in the sample holder and exposed to an external heat flux of 35 kW/m^2^ generated by the cone heater. The fire hazard parameters were analyzed using MLCCalc 1.0.3 software (Fire Testing Technology Ltd., East Grinstead, UK).

During the test, the following parameters were recorded or calculated: time to ignition (t_i_), time to flame-out (t_f-o_), heat release rate (HRR, kW/m^2^), maximum heat release rate (HRR_MAX_, kW/m^2^), time to maximum heat release rate (tHRR_MAX_, s), total heat release (THR, MJ/m^2^), average effective heat of combustion (EHC, MJ/kg), maximum effective heat of combustion (EHC_MAX_, MJ/kg), mass loss rate (MLR, g/s), maximum mass loss rate (MLR_MAX_, g/s), average mass loss rate (AMLR, g/m^2^s), fire growth rate index (FIGRA, kW/m^2^s), and maximum average rate of heat emission (MARHE, kW/m^2^).

#### 2.2.5. Smoke Optical Density

Smoke optical density was determined using a single-chamber smoke density test (SDC). The measurement procedure was carried out in accordance with PN-EN ISO 5659-2 [32]. Samples with dimensions of 75 mm × 75 mm × 5 mm were exposed to a heat flux of 25 kW/m^2^ generated by the cone heater. The measured parameters included the maximum smoke optical density (D_sMAX_), time to maximum smoke optical density (TD_sMAX_), smoke optical density after 4 min of testing (Ds(4)), and the area under the smoke emission curve during the first 4 min of the test (VOF4). These parameters were recorded using SDCSoft 1.0.4.0 software, Fire Testing Technology Ltd. (East Grinstead, UK). It should be noted that all recorded values are dimensionless, except for Ds(4), which is expressed in seconds.

#### 2.2.6. Toxicity

The toxicity of gaseous degradation products released from the investigated materials was analyzed using a coupled analytical system consisting of an Omega 5 gas analyzer, Bruker (Billerica, MA, USA), equipped with an FTIR spectrophotometer and coupled with an F1 Libra 209 thermogravimetric analyzer, Netzsch (Selb, Germany), hereafter referred to as the TG–gas analyzer system.

Thermal decomposition was performed using samples with a mass of 10 ± 1 mg at three temperatures: 450, 550, and 750 °C. The measurements were carried out in a synthetic air atmosphere with nitrogen/oxygen flow rates of 40/20 µL/min, respectively. The temperature programs were set to collect gaseous decomposition products for 30 min, including a 15 min isothermal segment at each selected temperature.

The gaseous products generated during thermal decomposition were analyzed in real time by FTIR spectroscopy, which enabled gas evolution to be monitored as a function of temperature. Spectra were recorded at intervals of 7–8 s, using 10 scans per spectrum, with a spectral resolution of 4 cm^−1^. Measurements were performed in transmission mode in the mid-infrared region from 4500 to 400 cm^−1^. The obtained spectral data were processed using OPUS GA 5.2.11.9 software (Bruker, Billerica, MA, USA), in which absorbance values were converted into gas concentrations expressed in ppm [33].

## 3. Results

### 3.1. Analysis of Chemically Modified Cotton

#### 3.1.1. FTIR Analysis of Chemical-Modified Cotton

**Figure 1:**
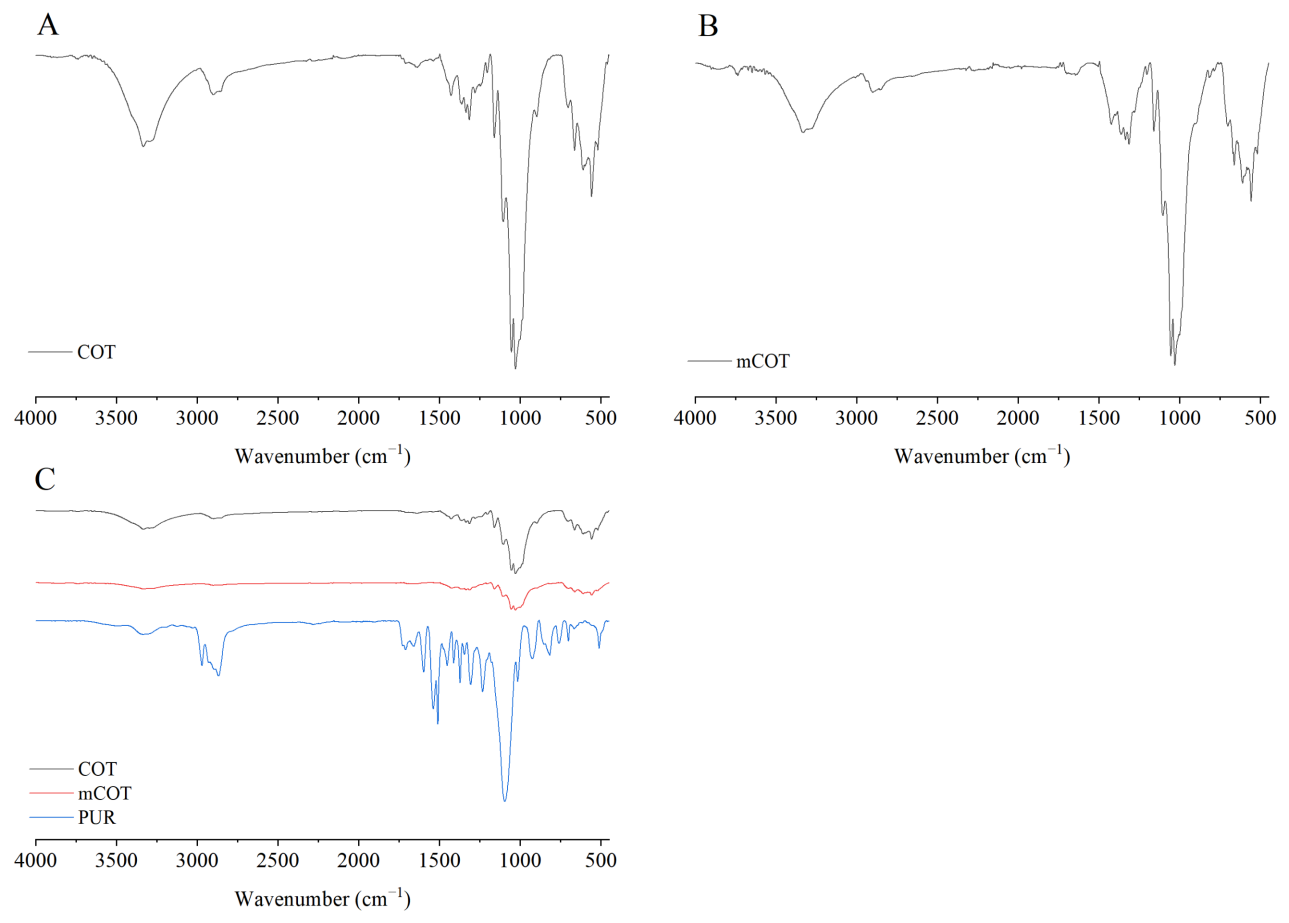
FTIR spectra of (**A**) unmodified cotton and (**B**) modified cotton; (**C**) comparison of cotton, modified cotton, and PUR.

An analysis of the FTIR spectra of cotton and chemically modified cotton (Figure 1) did not reveal the appearance of new absorption bands or distinct deformation of the bands characteristic of cellulose. This suggests that the chemical modification did not lead to the formation of new chemical bonds that could be unequivocally confirmed by FT-IR and that its effect may have been mainly surface-related.

#### 3.1.2. Surface Morphology Analysis of Chemical-Modified Cotton

**Figure 2:**
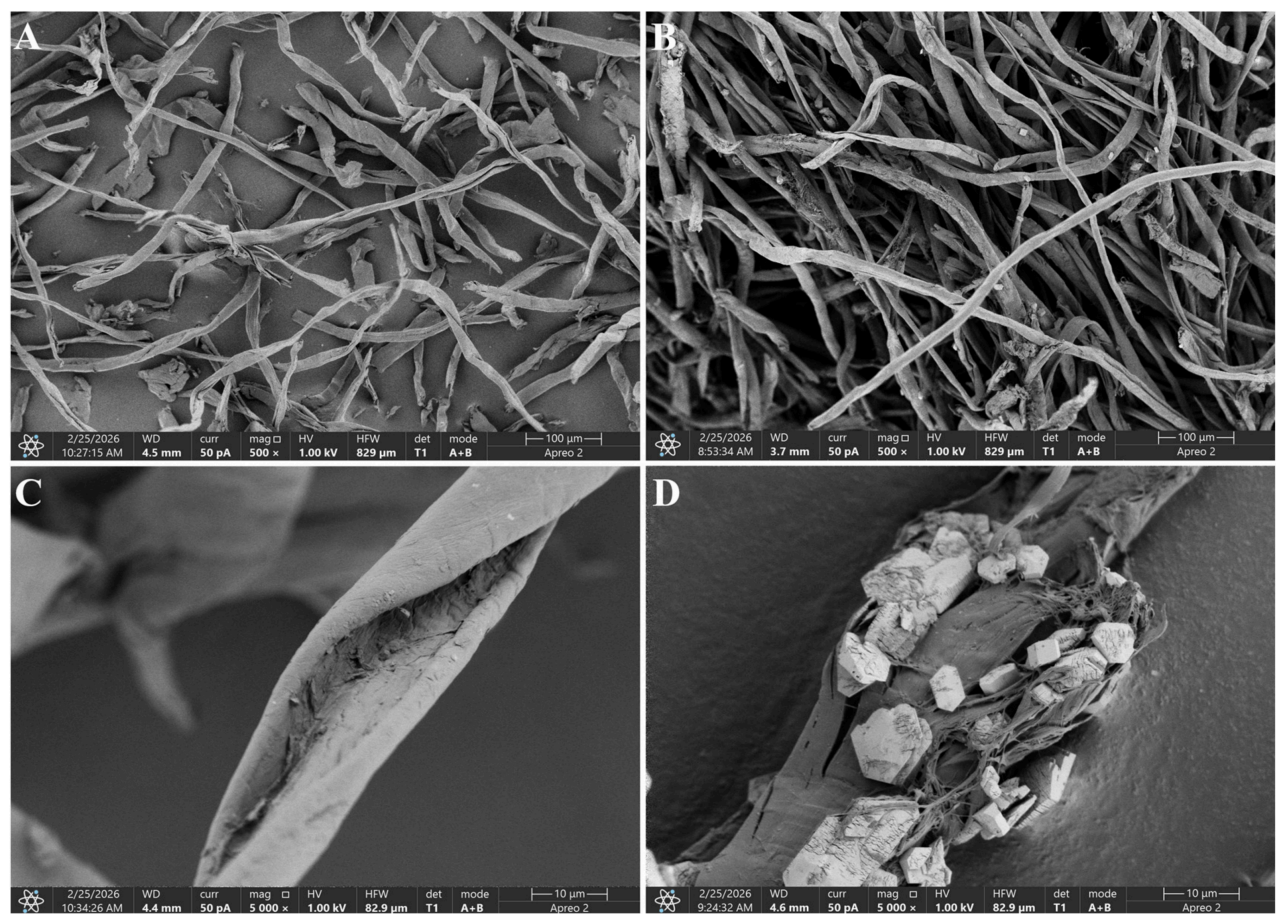
SEM images of (**A**) unmodified cotton, (**B**) chemically modified cotton, (**C**) unmodified cotton, and (**D**) chemically modified cotton. Scale: (**A**,**B**) 100 µm; (**C**,**D**) 10 µm.

SEM analysis (Figure 2) revealed the presence of particles deposited on the surface of chemically modified cotton fibres, indicating the effective functionalization process. These observations, combined with EDS analysis confirming the presence of elements characteristic of the applied modifying system, may confirm the deposition of the modifying layer on the fiber surface.

#### 3.1.3. Thermal Analysis of Chemical-Modified Cotton

**Figure 3:**
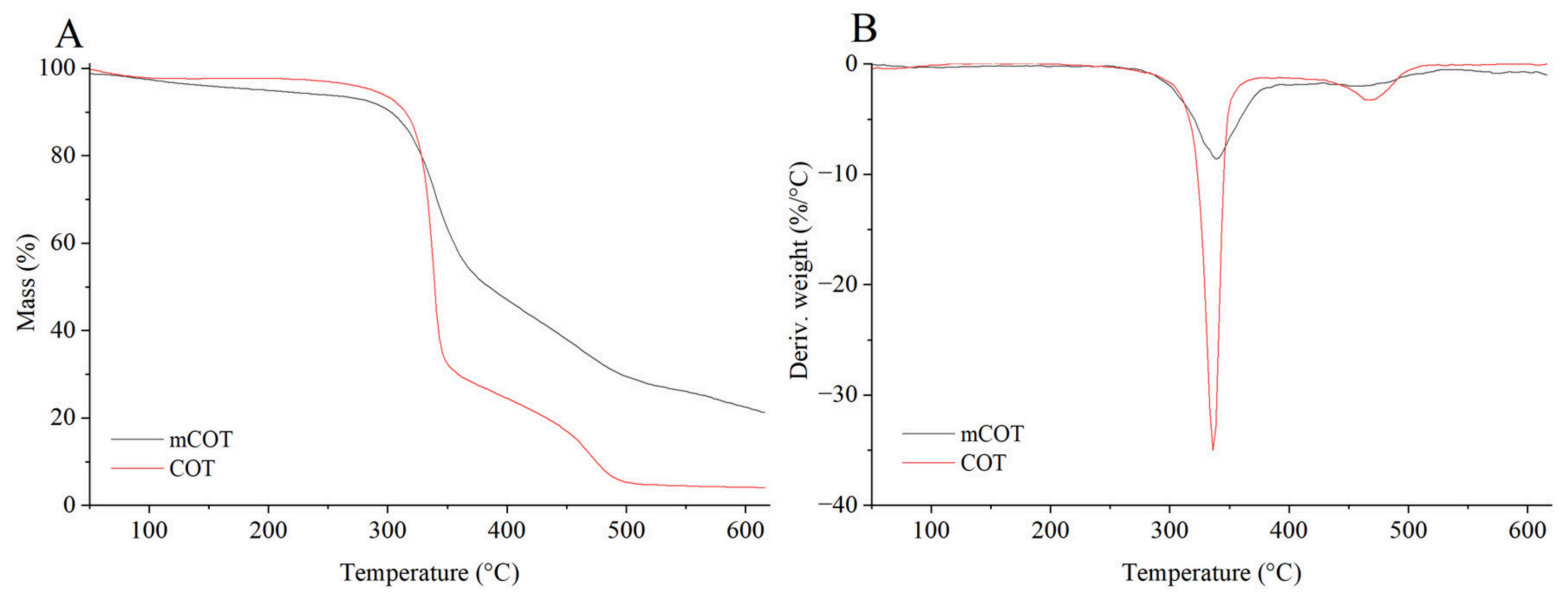
(**A**,**B**) TG and DTG curves of cotton and chemically modified cotton.

The final stage in evaluating the effectiveness of the modification was the thermal analysis of cotton before and after chemical functionalization. The TG curve of unmodified cotton (Figure 3A) indicates a two-step course of thermal degradation, which is confirmed by the DTG curve (Figure 3B). In the case of chemically modified cotton, a one-step thermal degradation process was observed, accompanied by an earlier mass loss and a marked increase in the residue after thermal decomposition.

The decrease in the maximum mass loss rate observed in the DTG curve indicates that the modification reduced the intensity of thermal degradation of the cellulose fibres. This effect may be associated with the presence of the modifying layer, which promotes the formation of a more stable residue in the condensed phase. The increased amount of residue after thermal decomposition provides an important argument confirming the effectiveness of the chemical modification of cotton and its potential usefulness as a component supporting the formation of a protective layer in PUR composites.

### 3.2. Surface Morphology Analysis Using SEM

The analysis of surface morphology and cellular structure is one of the key aspects in the characterization of polyurethane composites, particularly porous materials such as flexible PUR foams. In this type of material, the chemical composition and the size and shape of the cells, their degree of openness or closure, the thickness of the cell walls, and the homogeneity of additive distribution within the matrix are of considerable importance. These parameters may significantly affect the functional properties of foams, as well as their behaviour during exposure to elevated temperature and an external ignition source. Studies on fire-resistant polyurethane materials indicate that the modification of the cellular structure after the incorporation of flame-retardant additives is one of the factors contributing to improved fire safety. A decrease in the porosity of PUR composites reduces the amount of air physically entrapped within the matrix, which consequently leads to a pronounced reduction in both the combustion temperature of the composite and the rate of flame propagation into the interior of the PUR material. Moreover, the homogeneous dispersion of mineral or fibrous fillers in the polymer matrix promotes the formation of a uniform insulating interfacial layer, which hinders mass and energy transfer between the sample and the flame.

**Figure 4:**
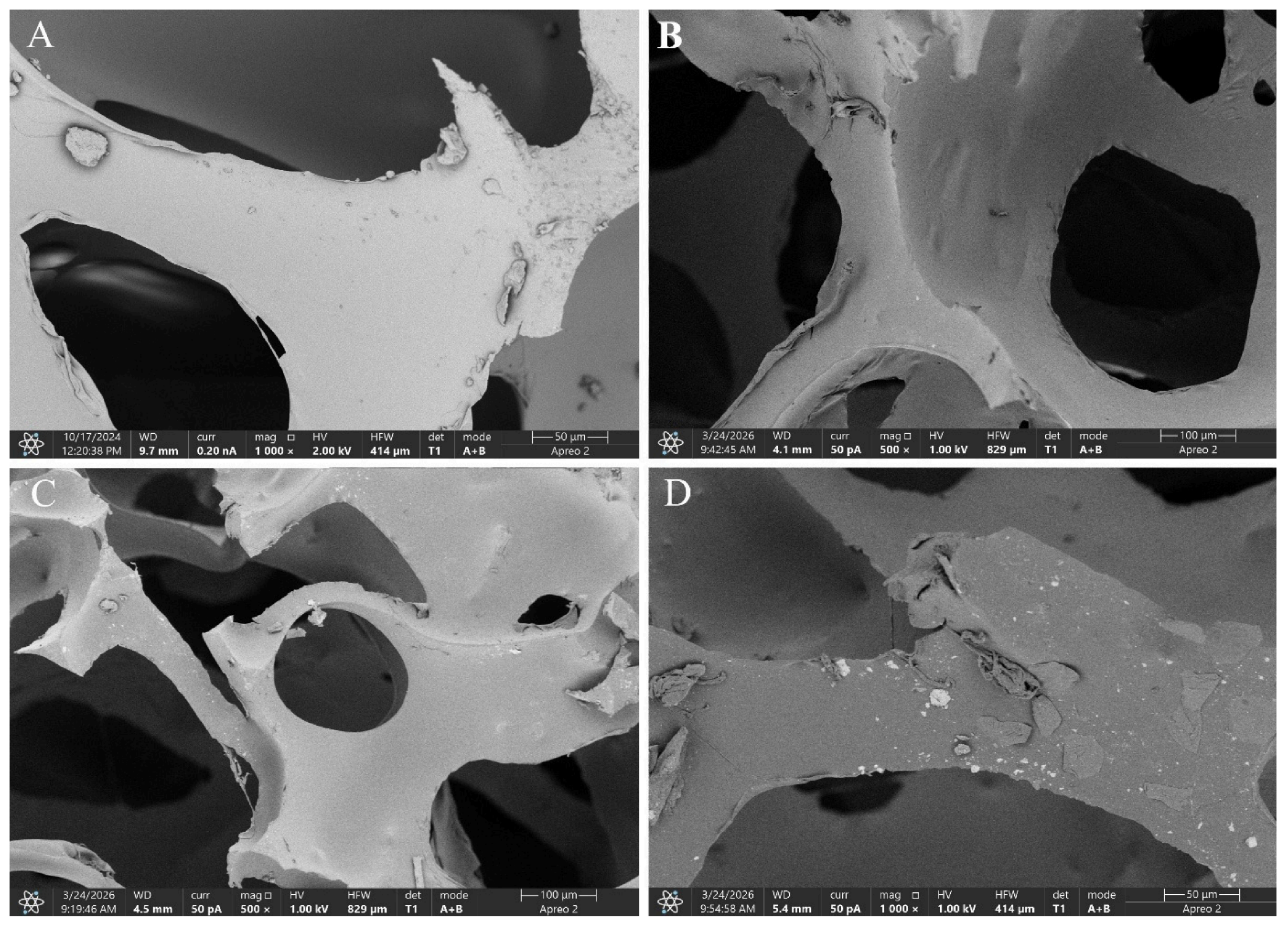
SEM imaging: (**A**) unmodified PUR foam, (**B**) PUR composite containing unmodified cotton, (**C**) PUR composite containing chemically modified cotton, (**D**) PUR composite containing chemically modified cotton and MPP.

Based on the SEM results, it can be clearly concluded that the incorporation of cotton fibres into the PUR matrix does not substantially affect the reduction in the porosity of the investigated composites (Figure 4A–C). In contrast, for the cotton–MPP system, a distinct reduction in composite porosity is clearly observed. In the case of the PUR-mCOT-MPP composite (Figure 4D), MPP particles dispersed within the matrix and on the PUR surface are visible. It should be emphasized that, on the one hand, the reduction in PUR porosity favourably affects its fire hazard parameters; on the other hand, it leads to a deterioration in flexibility, which is an important functional property of the polyurethane foam itself.

**Figure 5:**
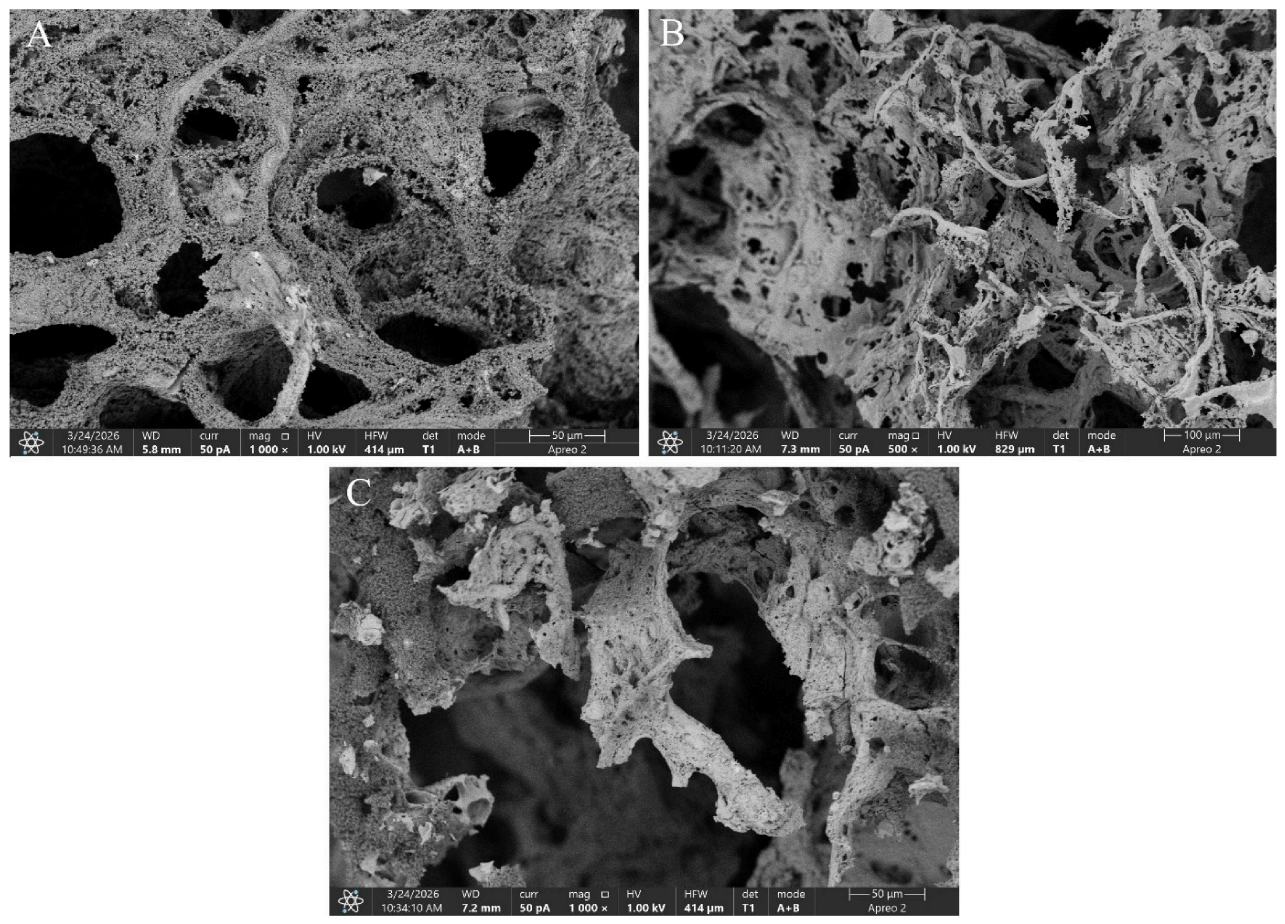
Combustion residues of: (**A**) PUR containing unmodified cotton, (**B**) PUR containing chemically modified cotton, and (**C**) PUR containing chemically modified cotton and MPP.

Using SEM, particularly interesting results were also obtained for the combustion residues of the investigated polyurethane foams (Figure 5A–C).

Based on the results presented in Figure 5, it was unexpectedly found that the combustion residue of the PUR-COT composite consisted of a three-dimensional network of cotton fibres. It should be clearly emphasized that the unfilled PUR composite underwent almost complete combustion; therefore, no combustion residue of the neat PUR foam is shown in Figure 5. The three-dimensional network of cotton fibres formed after the thermal decomposition of the PUR-COT composite indicates the good dispersion of the cotton fibres within the PUR matrix (Figure 5A). At the decomposition temperature of the PUR composite, the cotton fibres also undergo combustion; however, they do not disintegrate (Figure 5A–C). The resulting three-dimensional spatial network composed of carbonized cotton fibres (Figure 5A) undoubtedly improves the homogeneity and insulating capacity of the interfacial barrier layer, thereby limiting mass and energy transfer between the sample and the flame and, consequently, contributing to a reduction in the rate of thermal decomposition and combustion of PUR. Figure 5B shows the combustion residue of the PUR composite containing cotton modified with boron compounds, denoted as PUR-mCOT. In comparison with the combustion residue of the unmodified fibres shown in Figure 5A, the residue formed from the boron-modified fibres is markedly more homogeneous. This is primarily attributed to the formation of a ceramic-like coating on the fibre surface, which results in greater structural integrity of the three-dimensional network and, above all, enhanced resistance to elevated temperature and flame exposure compared with the network formed from unmodified cotton. In the case of the PUR-mCOT-MPP composite, the ceramizations of cellulose fibres were observed to be bonded together by MPP (Figure 5C).

**Figure 6:**
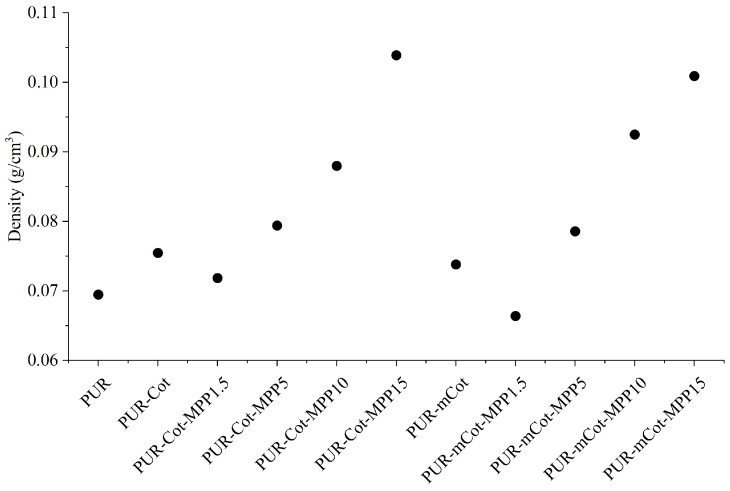
Apparent density of investigated PUR composites.

The results presented in Figure 6 indicate an increase in the apparent density of composites containing cotton fibres and MPP. The reference PUR foam exhibited an apparent density of 0.069 g/cm^3^. The incorporation of unmodified cotton increased this parameter to 0.075 g/cm^3^, whereas the use of cotton modified with boron compounds increased the density to 0.074 g/cm^3^. The introduction of MPP into the PUR matrix led to a pronounced increase in the apparent density of the investigated PUR composites.

Samples containing both cotton and MPP showed an increase in apparent density proportional to the MPP content in the polymer matrix (Figure 6).

The obtained results indicate that the presence of cotton fibres and MPP affects the foaming process, leading to changes in the organization of the cellular structure of the material. The increase in apparent density may indicate greater pore irregularity, a reduction in pore volume, and partial densification of the composite structure. This effect becomes more pronounced with increasing MPP content, suggesting that this additive, in combination with the cotton component, significantly influences the porous morphology of the resulting foams.

### 3.3. FTIR Analysis

The FTIR analysis of the composites revealed characteristic absorption bands corresponding to the typical bonds and functional groups that define polyurethane materials (Table 2).

In the range of 3370–3230 cm^−1^, a broad band assigned to the N-H stretching vibrations of urethane groups was observed. The presence and intensity of the bands located near 2265 and 3400 cm^−1^ are associated with unreacted NCO and OH groups, respectively. In the recorded spectrum, the high intensity of the band in the 3370–3230 cm^−1^ region indicates the presence of amide-related absorption associated with the N-H stretching vibrations of urethane moieties. Another prominent band detected in the PUR foam spectrum was located at 1710 cm^−1^ and was attributed to the stretching vibrations of the urethane carbonyl group involved in hydrogen bonding. The band observed at 1655 cm^−1^ was assigned to the carbamate group (C-H-N). The presence of these bands, together with the low intensity of the signal at 2265 cm^−1^, indicates that nearly all diisocyanate groups reacted during polymerization, leading to the formation of urethane linkages and amide-related structures. The band recorded at 1092 cm^−1^, assigned to the stretching vibrations of ether C-O-C bonds, confirms the presence of polyether segments in the polyurethane structure derived from the polyol used in the synthesis. In turn, the band at 1233 cm^−1^ is related to the vibrations of C-O groups. The absorption band at 1534 cm^−1^ corresponds to the C-N stretching vibrations and N-H bending vibrations of the amide II region. The bands observed at 1411 and 1597 cm^−1^ are characteristic of aromatic ring vibrations originating from the diisocyanate component, namely MDI.

The FTIR spectrum also showed bands at 2971 and 2868 cm^−1^, which were assigned to the asymmetric and symmetric stretching vibrations of C-H bonds in CH2 groups. In addition, the band at 1373 cm^−1^ is characteristic of C-H bending vibrations in CH2 groups.

**Figure 7:**
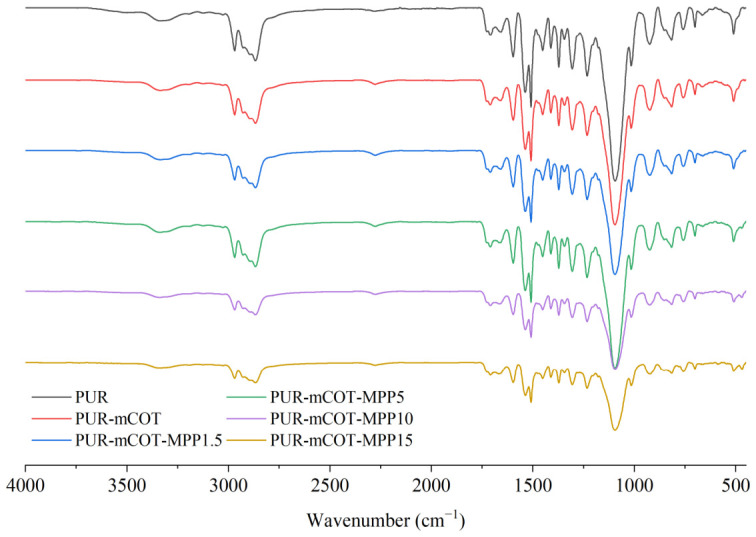
FTIR spectra of PUR composites.

The incorporation of cotton fibres, including chemically modified cotton, as well as MPP, did not affect the shape of the FTIR spectra (Figure 7).

### 3.4. Thermal Analysis for PUR Composites 

**Figure 8:**
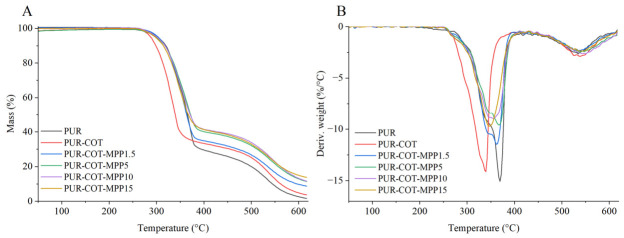
Thermal curves of PUR composites containing unmodified cotton: (**A**) TG curves, (**B**) DTG curves.

**Figure 9:**
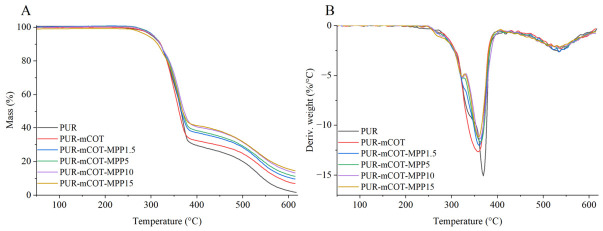
Thermal curves of PUR composites containing chemically modified cotton: (**A**) TG curves, (**B**) DTG curves.

The investigated PUR composites undergo one-step thermal decomposition within the temperature range of ΔT = 35–400 °C. The combustion of the residue formed after thermal decomposition occurs within the temperature range of ΔT = 400–600 °C (Figure 8 and Figure 9). The incorporation of both unmodified and chemically modified cotton, also in combination with MPP, has practically no effect on thermal parameters such as T_5_, T_50_, and T_RMAX_. Among the analyzed materials, the greatest decrease in the onset of thermal decomposition was observed for the PUR-Cot composite. This material show a shift in the T_5_ value towards lower temperatures, which may be attributed to the earlier thermal decomposition of cotton fibres, beginning at approximately 285 °C. At the same time, the PUR-COT composite did not exhibit a significant reduction in the dm/dt value, indicating that the presence of unmodified cotton did not limit the degradation rate of the PUR matrix. The obtained results suggest that cotton alone does not improve the thermal stability of PUR foam, and its presence may reduce the material’s resistance to the initial stage of thermal degradation. However, cotton, particularly chemically modified cotton, clearly affects the thermal decomposition rate, dm/dt, as well as the residue after thermal decomposition, expressed as P_TD_, and the residue at 600 °C, expressed as P_600_.

From the perspective of fire hazard, the reduction in dm/dt is of particular importance. The lower the value of this parameter, the smaller the amount of high-energy flammable degradation products transferred from the sample into the flame. Chemically modified cotton significantly decreases the thermal decomposition rate of PUR composites and also increases the residue after thermal decomposition, (P_TD_), as well as the residue remaining after decomposition at 600 °C (P_600_). The reduction in dm/dt and the increase in P_TD_ and P_600_ values result directly from the formation, during the thermal decomposition of the PUR composite, of a three-dimensional ceramic-like spatial network. By hindering mass transfer to the flame, this network promotes cyclization and carbonization processes in the residue formed after thermal decomposition (Table 3).

**Scheme 1:**
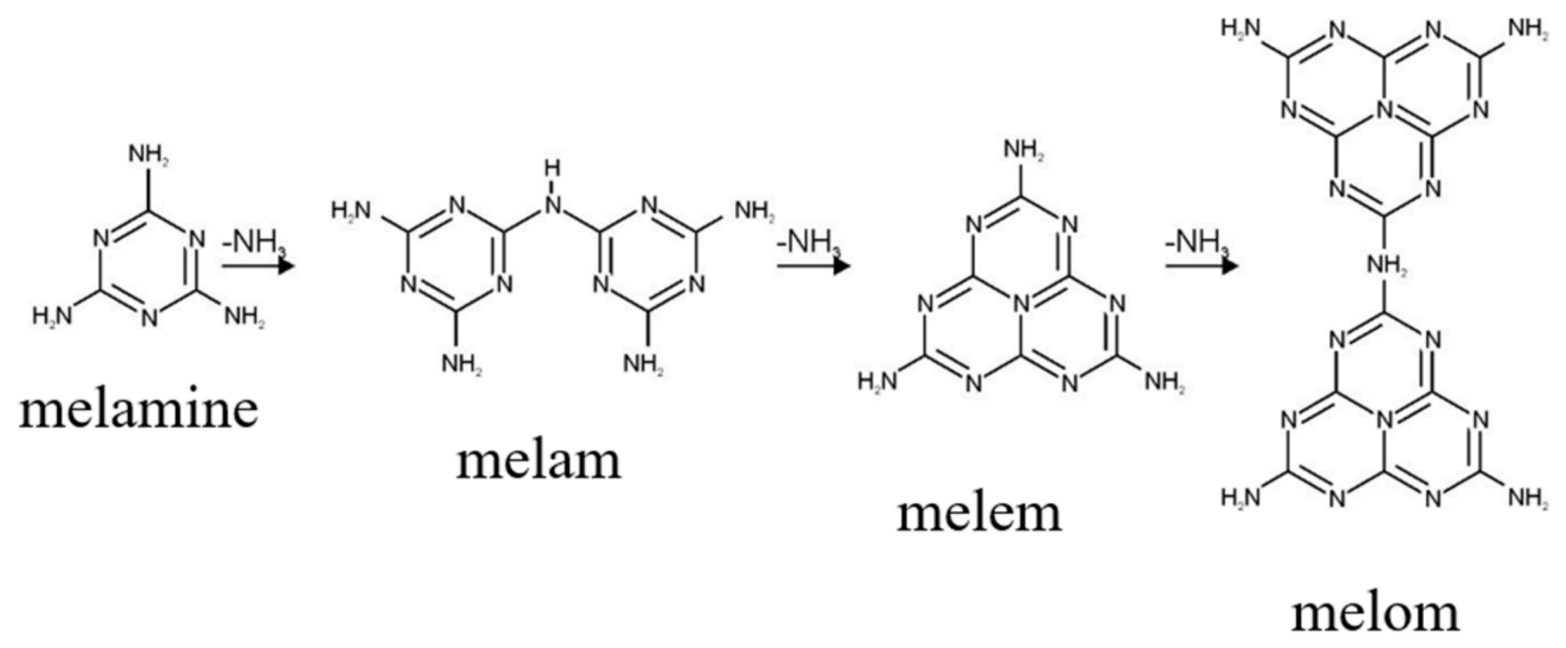
Thermal decomposition products of melamine to melam, melem and melon.

The incorporation of melamine polyphosphate (MPP) into the polymer matrix clearly increases both the residue after thermal decomposition and the combustion residue, i.e., the residue remaining at T = 600 °C. The flame-retardant activity of melamine polyphosphate is associated with its endothermic decomposition, which provides a cooling effect for the sample, the release of non-flammable ammonia, and the formation of condensation products such as melam, melem, and melon. The presence of these species in the interfacial layer formed during the combustion of the elastomeric material hinders mass and energy transfer between the condensed and gas phases (Scheme 1).

During the decomposition of melamine polyphosphate, acids such as HNO_2_ and HNO_3_ may also be formed, whose presence causes polymer decomposition to proceed according to an ionic mechanism. It cannot be excluded that the decomposition products of the flame retardant, after entering the flame zone, also reduce the rate of free-radical combustion processes. The decomposition products of melamine polyphosphate, namely melam, melem, and melon, may adsorb onto the surface of ceramized cotton fibres in the m-COT composites, thereby increasing the insulating efficiency and homogeneity of the interfacial layer (Table 3).

### 3.5. Flammability Evaluated by Cone Calorimetry

The flammability of flexible PUR foams is one of the key parameters determining their safe use under conditions involving flame exposure and elevated temperature. In fire hazard assessment, parameters such as the HRR, THR, FIGRA, and MARHE are of particular importance, as they enable a comprehensive evaluation of combustion intensity, fire growth dynamics, and the total amount of heat released.

**Figure 10:**
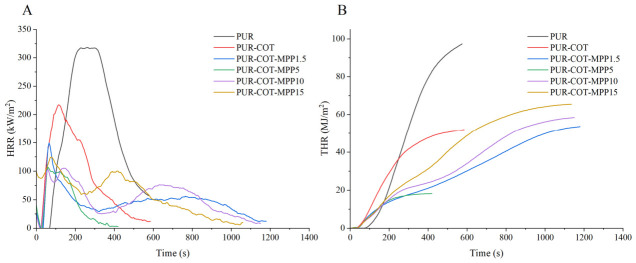
Flammability test results for PUR-COT composites: (**A**) HRR curves and (**B**) THR curves.

The results obtained by cone calorimetry indicate that the reference PUR sample exhibits the highest values of HRR_MAX_ and MARHE (Table 4, Figure 10A). The incorporation of unmodified cotton into the PUR matrix results in a marked reduction in the fire hazard parameters of the PUR-COT composite (Table 4). For the PUR-COT sample, HRR_MAX_ decreased by 31.7%, whereas the MARHE was reduced by as much as 67.7% compared with the reference sample. A substantially reduced THR value was also observed for the PUR foam containing cotton fibres (Table 4, Figure 10B). The lower THR value of the PUR-COT composite compared with the reference sample clearly indicates that cotton effectively suppresses the exothermic thermal decomposition and combustion reactions occurring in the investigated sample.

**Figure 11:**
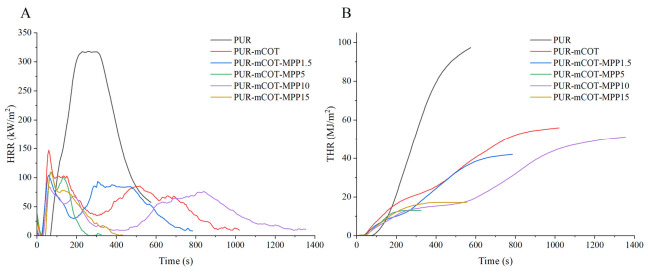
Flammability test results for PUR-mCOT composites: (**A**) HRR curves and (**B**) THR curve.

The cone calorimetry results clearly indicate that, in the presence of chemically modified cotton (Table 5, Figure 11A,B), the fire hazard parameters of PUR, namely the HRR, HRC, THR, FIGRA, and MARHE, are reduced to an even greater extent than in the case of unmodified cotton. The reduction in flammability observed for both the PUR-COT and PUR-mCOT composites results from the formation, during the thermal decomposition of PUR, of a three-dimensional carbon fibre network that stabilizes the intumescent layer at the sample–flame interface (Figure 5A). During the thermal decomposition and combustion of PUR-mCOT composites, the resulting spatial network exhibits a hybrid structure, in which a carbonaceous core is covered with a ceramic-like coating, which favourably affects both the homogeneity and the insulating efficiency of the interfacial layer formed during the combustion of the PUR composites (Figure 5B). The incorporation of melamine polyphosphate into the PUR-COT and PUR-mCOT composite matrices results in a further reduction in the flammability of the investigated PUR materials. The flame-retardant action of MPP is associated both with the release of non-flammable ammonia, which dilutes combustible gases and thereby reduces the efficiency of high-energy gas phase reactions, and with the formation of cyclic condensation products such as melon, which stabilize the interfacial barrier layer.

**Figure 12:**
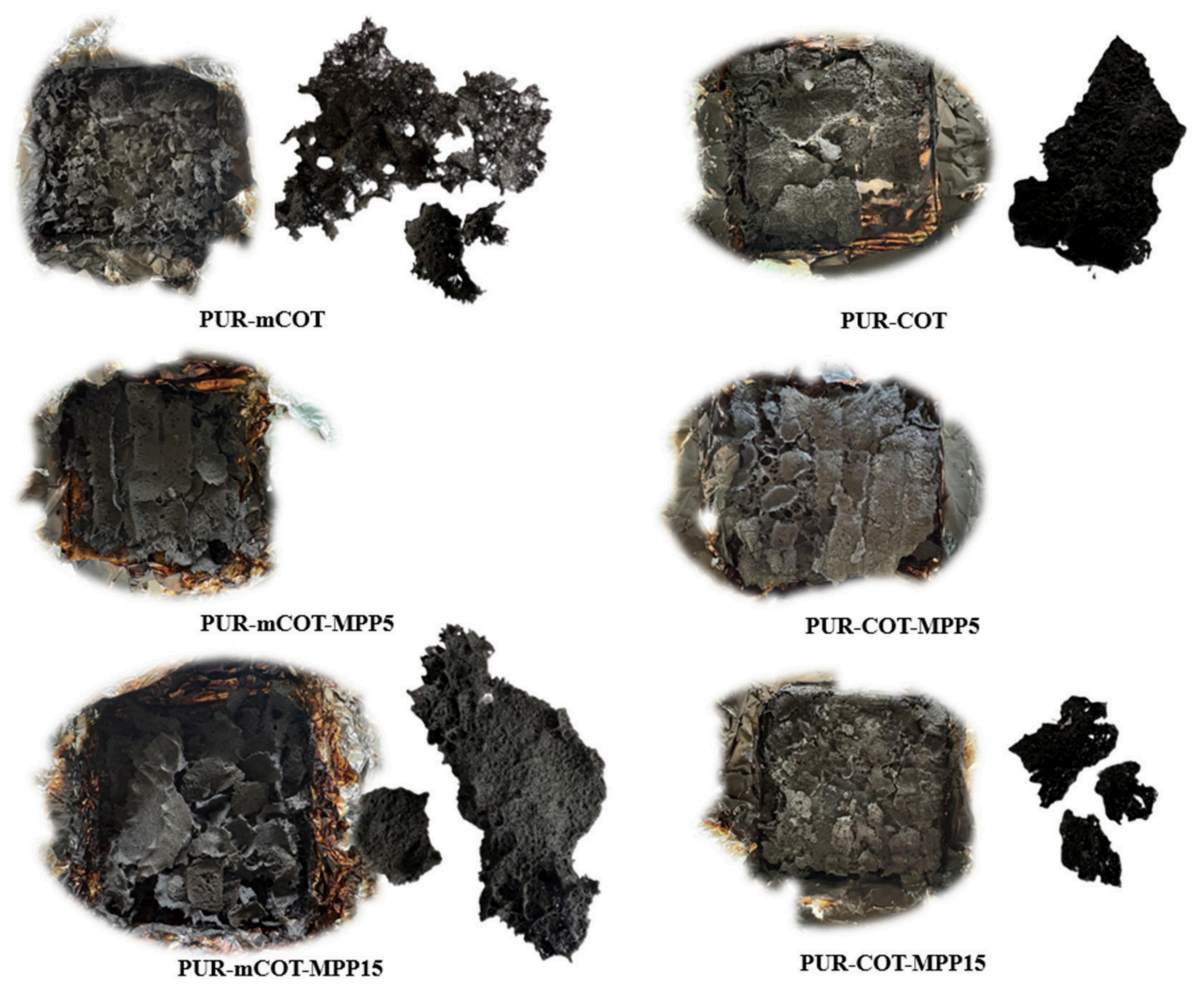
Combustion residue of PUR composites.

The obtained results confirm that the use of cotton chemically modified with boron compounds, particularly in combination with MPP, leads to a distinct improvement in the fire resistance of flexible PUR foams (Figure 12). This effect may be attributed to the intensified formation of a protective barrier layer, reduced release of flammable degradation products, and an increased amount of solid residue, which collectively result in lower combustion intensity and a reduced fire hazard of the investigated composites.

### 3.6. Smoke Optical Density for PUR Composites

The assessment of the fire hazard of polymeric materials is not limited solely to the analysis of their flammability and heat release parameters but also includes the characterization of smoke emission, which is one of the key factors determining safety under fire conditions. Smoke is defined as an aerosol composed of solid and liquid particles generated during incomplete combustion and pyrolysis. It contains, among others, soot particles, condensation products, liquid mists, and a mixture of volatile organic and inorganic compounds. Depending on the chemical composition of the material and the combustion conditions, the emitted components may exhibit toxic, irritant, or corrosive effects. The intensity of smoke emission and the mechanism of smoke formation are strongly dependent on numerous factors, such as the chemical structure of the polymer, the carbon content in the material structure, the degree of aromaticity, the presence of flame-retardant additives, and the course of thermal degradation and combustion processes. A high carbon content in the material may promote the formation of condensed products in the gas phase, which consequently leads to increased smoke emission.

**Figure 13:**
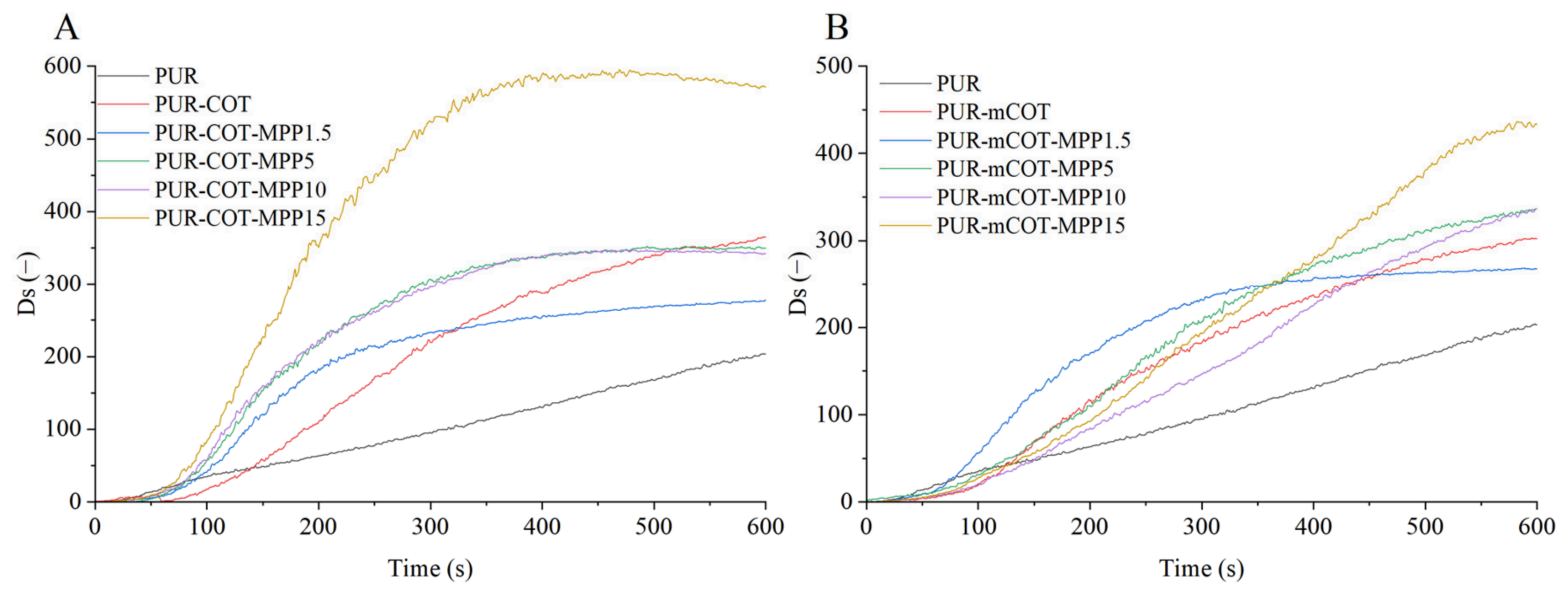
Optical smoke density curves of PUR composites: (**A**) PUR with unmodified cotton, (**B**) PUR with chemical-modified cotton.

The analysis of the obtained results (Table 6, Figure 13) indicates that the incorporation of both unmodified cotton and chemically modified cotton into the PUR matrix leads to an increase in the amount of smoke emitted.

Among all the analyzed systems, the highest smoke emission intensity was observed for the composites containing unmodified cotton and MPP. This is particularly well illustrated by the VOF4 parameter, which describes smoke emission intensity in the initial stage of combustion. For the PUR-Cot composite, the value of this parameter was 200.5, whereas for the PUR-Cot-MPP15 system it increased to as much as 683.8. These results indicate that the use of unmodified cotton in combination with increasing MPP content promoted the intensification of smoke-forming processes, most probably as a result of the enhanced formation of condensed residue and secondary pyrolysis products. It also cannot be excluded that inert gases released during MPP decomposition, such as ammonia, act as carriers for soot particles. Composites containing boron-modified cotton and MPP also exhibited increased smoke emission; however, the intensity of this phenomenon was distinctly lower than that observed for the corresponding systems containing unmodified cotton. For this group of materials, the VOF4 values ranged from 163.9 for the PUR-mCot-MPP10 sample to 350.3 for the PUR-mCot-MPP1.5 sample (Table 6, Figure 13). The increase in optical smoke density in the composites, probably resulting from a higher proportion of the carbonaceous phase and more intensive formation of smoke products, may constitute a significant evacuation hazard due to reduced visibility in the fire zone. Therefore, in the next stage of this study, the toxicity of gaseous products released during the thermal decomposition and combustion of the developed PUR composites was analyzed.

### 3.7. Toxicity of Gaseous Degradation Products

The most important gases posing a toxicological hazard include asphyxiant gases, such as CO and HCN. Moreover, these gases significantly reduce the possibility of safe evacuation during a fire. Their presence, even at relatively low concentrations, may lead to the rapid impairment of respiratory and neurological functions and, consequently, may pose an immediate threat to life. The results presented in Table 7 indicate that the applied flame-retardant systems significantly reduce the emission of HCN and CO, particularly at 550 °C, which is a critical temperature from the perspective of the generation of highly toxic decomposition products. At the higher temperature of 750 °C, HCN emission also remains lower than that recorded for the unmodified PUR foam; however, the degree of HCN reduction is less pronounced than at 550 °C. At the same time, a substantial reduction in CO emission is still observed at 750 °C, in many cases exceeding 50% relative to the reference sample. Despite the pronounced reduction in the emission of toxic decomposition products, higher gas emissions, mainly HCN, were observed at 750 °C for the PUR-Cot composite. The composites containing a low amount of MPP, i.e., 1.5%, also exhibited increased emission of this gas. These results confirm that the incorporation of MPP at 5 and 10% promotes the formation of the most stable protective layer, which limits the emission of toxic products during thermal decomposition.

The obtained results are confirmed by the toxicity index values, which showed that the incorporation of cotton and MPP into the PUR matrix reduces the toxicity of the developed composites (Table 8).

A particularly favourable effect was observed for systems containing cotton modified with boron compounds in combination with MPP, for which the WLC_50SM_ values ranged from 5.3 to 8.2. These results therefore indicate that the applied modifying system effectively limits the emission of the most hazardous gaseous products of thermal decomposition and combustion, thereby reducing the overall toxicological hazard of the investigated materials (Table 8).

**Figure 14:**
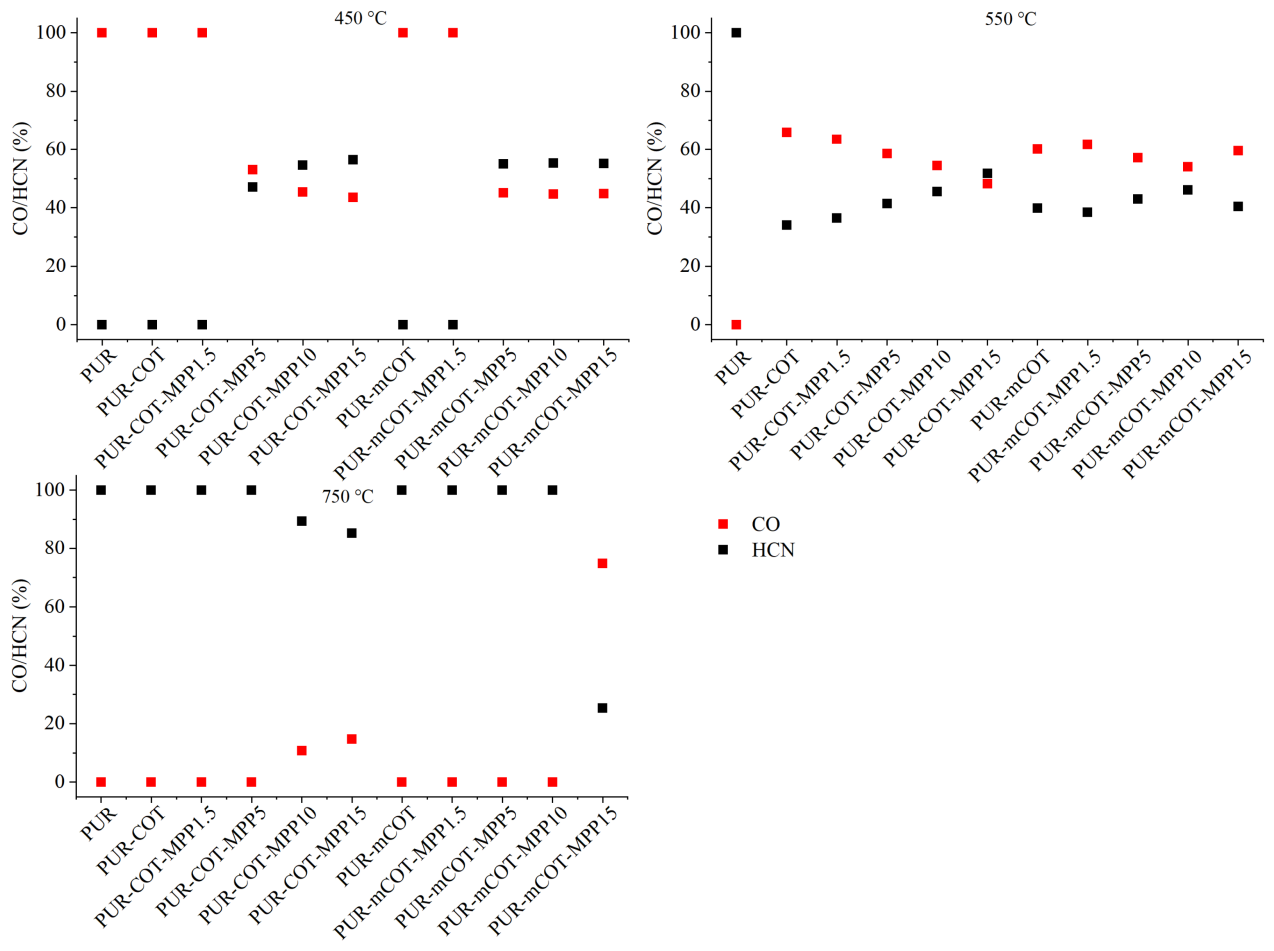
Percentage relationship between CO and HCN during thermal decomposition of composites.

For the assessment of gaseous degradation products generated during the thermal decomposition of PUR materials, the CO/HCN ratio is a highly useful parameter. This parameter reflects the percentage contribution of both gases to the overall toxic effect, with HCN being of greater toxicological significance due to its substantially higher unit toxicity compared with CO (Figure 14). The analysis of the results showed that at T = 450 °C, CO was the dominant contributor in the CO/HCN relationship. At T = 550 °C, CO also remained the dominant component of the CO/HCN mixture for most of the composites. The exceptions were the PUR-COT-MPP15 and PUR-mCOT-MPP10 composites, for which the contributions of both gases were comparable and amounted to approximately 50/50% (Figure 14). The highest relative contribution of HCN with respect to CO was observed for all investigated composites at 750 °C. This effect is associated with the complete thermal decomposition of nitrogen-containing segments characteristic of polyurethane materials and melamine-based additives.

## 4. Conclusions

This study investigated the effects of cotton chemically modified with boron compounds, also in a synergistic system with melamine polyphosphate (MPP), on the thermal properties, flammability, smoke emission, and toxicity of thermal decomposition and combustion products of flexible PUR foams. The results demonstrated that the incorporation of an appropriate ratio of fillers into the polyurethane matrix leads to a significant reduction in the fire hazard of PUR materials. Surface morphology analysis after cone calorimetry revealed that the presence of chemically modified cotton promotes the formation of a three-dimensional spatial network of carbonized cotton fibres. During the decomposition of the PUR composite, the cotton fibres did not disintegrate; instead, the resulting three-dimensional network composed of carbonized fibres positively affected the homogeneity and insulating efficiency of the interfacial barrier layer, limiting mass and energy transfer between the sample and the flame.

This effect was reflected in the flammability results, which showed that both chemically modified cotton alone and its combination with MPP reduced HRR_MAX_, THR, and MARHE values compared with the unmodified reference foam. However, the formation of a carbonaceous protective layer led to a considerable increase in smoke optical density. Nevertheless, the analysis of gaseous degradation products generated during the thermal decomposition of the composites showed that CO_2_ was the main component responsible for the increased smoke emission, whereas the concentrations of HCN and CO were substantially reduced.

In summary, new polyurethane–cotton composites were developed, in which chemically modified cotton acts as both a filler and a carrier for flame retardants. In combination with melamine polyphosphate, it also functions as a stabilizing component of the interfacial barrier layer formed during combustion.

## Figures and Tables

**Table 1:** Composition of PUR–cotton composites, expressed in parts by weight.

Composite	Cotton (Cot)	Modified Cotton (mCot)	MPP
PUR-COT	5	-	-
PUR-COT-MPP1.5	5	-	1.5
PUR-COT-MPP5	5	-	5
PUR-COT-MPP10	5	-	10
PUR-COT-MPP15	5	-	15
PUR-mCOT	-	5	-
PUR-mCOT-MPP1.5	-	5	1.5
PUR-mCOT-MPP5	-	5	5
PUR-mCOT-MPP10	-	5	10
PUR-mCOT-MPP15	-	5	15

**Table 2:** FTIR band assignments of analyzed polyurethane materials [34,35,36].

No.	Wavenumber (cm^−1^)	Bond Assignment/Attributed Band
1.	3370–3230	N-H stretching vibrations of urethane groups
2.	2971	Asymmetric C-H stretching vibrations of CH_2_ groups
3.	2868	Symmetric C-H stretching vibrations of CH_2_ groups
4.	2265	Asymmetric stretching vibrations of the isocyanate group N=C=O
5.	1710	Stretching vibrations of the urethane carbonyl C=O group
6.	1655	Amide I band of the carbamate group, C-H-N
7.	1597	C=C vibrations of aromatic rings derived from MDI
8.	1538	Amide II band: C-N stretching vibrations and N-H bending vibrations
9.	1509	Amide II band: C-N deformation vibrations and N-H deformation vibrations
10.	1411	C-N stretching vibrations of the isocyanurate group
11.	1373	C-H bending vibrations of CH_2_ groups
12.	1233	C-O-C/C-O stretching vibrations of ether and urethane groups
13.	1094	C-O-C stretching vibrations characteristic of the polyol
14.	1016	C-H bending vibrations
15.	925	C-H bending vibrations
16.	819	C-H bending vibrations

**Table 3:** Thermal parameters of PUR composites.

Composite	T_5_ (°C)	T_50_ (°C)	T_RMAX_ (°C)	dm/dt (%/min)	P_TD_ (°C)	P_600_ (°C)
PUR	301	364	368	15	27.3	2.8
PUR-COT	287	339	339	14	27.4	4.9
PUR-COT-MPP1.5	302	363	362	11.5	31.6	9.8
PUR-COT-MPP5	300	370	367	9.6	33.2	13
PUR-COT-MPP10	301	370	357	9	38.6	13.3
PUR-COT-MPP15	297	366	347	9.7	39.5	15
PUR-mCOT	304	360	357	12.6	30.7	7.8
PUR-mCOT-MPP1.5	305	365	359	12	35.2	10.6
PUR-mCOT-MPP5	304	367	365	11.7	36.5	12.2
PUR-mCOT-MPP10	305	372	362	11.4	39	14.4
PUR-mCOT-MPP15	292	369	359	11.2	39.7	15.6

**Table 4:** Cone calorimetry results for PUR-COT composites.

Composite	PUR	PUR-COT	PUR-COT-MPP1.5	PUR-COT-MPP5	PUR-COT-MPP10	PUR-COT-MPP15
Initial mass	28	26.8	30.3	32.2	38.8	41.5
Mass lost	27	25.1	27	12	32.1	34.1
ti (s)	54	24	33	28	24	29
tf-o (s)	455	474	1092	294	1062	1008
HRRmax (kW/m^2^)	318	217.3	149.5	107	105	124.6
HRR (kW/m^2^)	218.9	111	49.2	64	55	65
EHCmax (MJ/kg)	79.35	54	74.7	76.5	60.6	80
EHC (MJ/kg)	29.71	18.3	17.5	13.4	16	17
THR (MJ/m^2^)	87.7	50	52.3	17.2	57.3	64.2
FIGRA (kW/m^2^·s)	1.20	1.9	2.3	1.8	0.75	0.8
MARHE (kW/m^2^)	198.36	146.8	73	74.6	76.9	85.43

**Table 5:** Cone calorimetry results for PUR-mCOT composites.

Composite	PUR	PUR-mCot	PUR-mCot-MPP1.5	PUR-mCot-MPP5	PUR-mCot-MPP10	PUR-mCot-MPP15
Initial mass	28	27.6	25.8	31.7	38.8	42.5
Mass lost	27	27.3	22.3	9.2	32	12.8
ti (s)	54	27	21	40	30	41
tf-o (s)	455	881	708	209	1295	426
HRRmax (kW/m^2^)	318	147.3	104.25	110.23	84	109.8
HRR (kW/m^2^)	218.9	63.2	60.1	71.76	40	44
EHCmax (MJ/kg)	79.35	58.5	55.7	72.4	76.5	32.3
EHC (MJ/kg)	29.71	18	17	13	14.3	12
THR (MJ/m^2^)	87.7	54.2	41.1	12.5	50.4	17.2
FIGRA (kW/m^2^·s)	1.20	2.5	1.7	1.6	1.3	1.6
MARHE (kW/m^2^)	198.36	83.6	65.3	65.6	53.3	61.2

**Table 6:** Optical smoke density analysis of PUR composites.

Composite	D_sMAX_	TD_sMAX_	Ds(4)	VOF4
PUR	203.8	598	74.22	173.8
PUR-COT	365	598	156	200.5
PUR-COT-MPP1.5	277.7	600	207.6	343
PUR-COT-MPP5	351.9	494	258.7	421.5
PUR-COT-MPP10	347.8	509	253.6	438.3
PUR-COT-MPP15	595.2	469	444.8	683.8
PUR-mCOT	302.8	595	146.2	208.3
PUR-mCOT-MPP1.5	268.4	589	199.1	350.3
PUR-mCOT-MPP5	336.3	600	151.5	336.3
PUR-mCOT-MPP10	336.6	599	108.7	163.9
PUR-mCOT-MPP15	436.1	583	131.3	191.9

**Table 7:** Maximum concentrations of gases released from PUR composites, expressed in mg/m^3^.

Composite	CO_2_	CO	HCN	NO_X_
450				
PUR	1232.7	1408.0	0.0	0.0
PUR-COT	760.1	318.3	0.0	0.0
PUR-COT-MPP1.5	745.2	470.0	0.0	0.0
PUR-COT-MPP5	945.1	393.5	50.4	0.0
PUR-COT-MPP10	970.9	299.8	55.5	0.0
PUR-COT-MPP15	915.7	253.8	69.8	0.0
PUR-mCOT	938.2	492.2	0.0	0.0
PUR-mCOT-MPP1.5	826.0	458.8	0.0	0.0
PUR-mCOT-MPP5	941.9	388.2	0.0	0.0
PUR-mCOT-MPP10	852.8	323.3	39.5	0.0
PUR-mCOT-MPP15	1086.4	291.2	67.2	0.0
550				
PUR	2530.9	2716.0	326.5	19.7
PUR-COT	1532.3	810.4	142.5	0.0
PUR-COT-MPP1.5	1924.2	894.1	169.8	6.7
PUR-COT-MPP5	2139.0	701.6	160.3	5.6
PUR-COT-MPP10	2313.0	638.9	172.0	0.0
PUR-COT-MPP15	2557.5	548.6	174.4	0.0
PUR-mCOT	1854.5	859.9	159.4	0.0
PUR-mCOT-MPP1.5	1997.6	996.5	193.4	0.0
PUR-mCOT-MPP5	1972.2	767.6	158.5	0.0
PUR-mCOT-MPP10	2273.3	699.3	176.3	0.0
PUR-mCOT-MPP15	2358.5	563.6	155.7	0.0
750				
PUR	3994.0	2673.1	267.0	54.5
PUR-COT	2179.6	1288.5	306.3	0.0
PUR-COT-MPP1.5	2076.2	1228.3	271.4	0.0
PUR-COT-MPP5	2680.4	1043.6	271.3	0.0
PUR-COT-MPP10	2795.8	827.4	221.7	0.0
PUR-COT-MPP15	3180.8	719.8	210.7	0.0
PUR-mCOT	2383.0	1158.0	190.5	0.0
PUR-mCOT-MPP1.5	2192.0	1244.4	266.8	0.0
PUR-mCOT-MPP5	2494.7	1071.9	267.6	0.0
PUR-mCOT-MPP10	2719.9	919.0	257.1	0.0
PUR-mCOT-MPP15	2656.3	671.4	202.8	0.0

**Table 8:** Toxicometric index.

Composite	WLC_50_ 450 °C	WLC_50_ 550 °C	WLC_50_ 750 °C	WLC_50SM_
PUR	14.8	0.2	0.5	5.2
PUR-COT	6.1	4.6	0.3	3.7
PUR-COT-MPP1.5	17.5	2.7	0.6	6.9
PUR-COT-MPP5	16.6	3.1	0.4	6.7
PUR-COT-MPP10	18.3	2.5	0.9	7.3
PUR-COT-MPP15	17.1	2.3	0.8	6.7
PUR-mCOT	17.6	4.4	2.5	8.2
PUR-mCOT-MPP1.5	13.9	1.7	0.6	5.4
PUR-mCOT-MPP5	15.9	3.2	0.5	6.5
PUR-mCOT-MPP10	16.2	2.3	0.6	6.4
PUR-mCOT-MPP15	16.4	3.8	1.4	7.2

## Data Availability

The original contributions presented in this study are included in the article. Further inquiries can be directed to the corresponding authors.
